# A novel FISH technique for labeling the chromosomes of dinoflagellates in suspension

**DOI:** 10.1371/journal.pone.0204382

**Published:** 2018-10-24

**Authors:** Rosa I. Figueroa, Alfredo de Bustos, Ángeles Cuadrado

**Affiliations:** 1 Instituto Español de Oceanografia (IEO), Subida a Radio Faro 50, Vigo, Spain; 2 Universidad de Alcala (UAH), Dpto Biomedicina y Biotecnología, Alcalá de Henares, Madrid, Spain; Politechnika Slaska, POLAND

## Abstract

Dinoflagellates possess some of the largest known genomes. However, the study of their chromosomes is complicated by their similar size and their inability to be distinguished by traditional banding techniques. Dinoflagellate chromosomes lack nucleosomes and are present in a liquid crystalline state. In addition, approaches such as fluorescent in situ hybridization (FISH) are problematic because chromosomes are difficult to isolate from the nuclear membrane, which in dinoflagellates remains intact, also during mitosis. Here we describe a novel, reliable and effective technique to study dinoflagellate chromosomes by physical mapping of repetitive DNA sequences in chromosomes in suspension (FISH-IS), rather than on a microscope slide. A suspension of non-fixed chromosomes was achieved by lysing the cells and destabilizing the nuclear envelope. This treatment resulted in the release of the permanently condensed chromosomes in a high-quality chromosomal suspension. Nevertheless, slide preparations of the chromosomes were not suitable for conventional FISH because the nuclear integrity and chromosomal morphology was destroyed. Our newly developed, simple and efficient FISH-IS technique employs fluorescently labeled, synthetic short sequence repeats that are hybridized with suspended, acetic-acid-pretreated chromosomes for 1 h at room temperature. The method can be successfully used to discriminate single chromosomes or specific chromosomal regions, depending on the specificity of the repeat sequences used as probes. The combination of FISH-IS and flow sorting will improve genomic studies of dinoflagellates, overcoming the difficulties posed by their huge genomes, including long stretches of non-coding sequences in multiple copies and the presence of high-copy-number tandem gene arrays.

## Introduction

Dinoflagellates are a large and diverse group of protists that encompasses extremely diverse organisms with photosynthetic, symbiotic, mixotrophic or parasitic life styles [1]. These organisms have already been studied extensively as primary producers, essential symbionts of reef-building corals and as producers of neurotoxins and other toxic metabolites with important health and ecological impacts. Nonetheless, although sequence information for dinoflagellates is available, their enormous genomes have hindered both efforts to obtain a reference sequenced genome and detailed genomic studies of dinoflagellates [2, 3]. In fact, the genomes of some dinoflagellates are the largest known among eukaryotes (up to 245 giga bp (GB) of DNA) and are ~80 times larger than the human genome [4]. Moreover, while the dinoflagellate genome is in general structured over hundreds of chromosomes, both histones and the typical nucleosomal organization are lacking [5]. Instead, dinoflagellate chromosomes exist in a cholesteric liquid crystal state, as seen under polarized light, and have an arched fibrillary appearance on electron micrographs. Divalent cations and HCc proteins, a family of DNA-binding proteins with homologies to bacterial and eukaryotic histone H1, are involved in chromatin compaction [6]. Another unique feature is that dinoflagellate chromosomes remain condensed throughout the cell cycle and are thus permanently visible but without Q, G or C banding. Mitosis is also distinctive, as it does not include either the breakdown of the nuclear envelope (endomitosis) [7] or a typical metaphase plate, such that chromosomal alignment is absent. Based on these features dinoflagellates constitute a unique biological model for the study of genome organization and expression [8] and an extremely interesting model for evolutionary comparisons in the rising field of the 4D nucleome research [9]. However, the protocols used in investigations of typical eukaryotes must be modified substantially [10], as in dinoflagellates they do not suffice to provide clear and identifiable mitotic chromosomal spreads for optical microscopy [11]. Additional challenges to studies of the organization, function and evolution of dinoflagellate chromosomes relate to the difficulty in culturing dinoflagellates in vitro.

In recent decades, the use of fluorescent in situ hybridization (FISH) to reveal a broad range of properties of nuclei and chromosomes in both animals and plants has become routine in research laboratories throughout the world. In dinoflagellates, FISH analyses have been employed to characterize the telomeres of a few species [12, 13] and the 45S rDNA locus in species of the genera *Alexandrium* and *Karenia* [14, 15]. However, because the permanently intact nuclear envelope of dinoflagellates prevents chromosome separation, even during mitosis, their chromosomes cannot be readily extruded using conventional methods (i.e., squashing, dropping) and chromosomal preparations suitable for epifluorescence microscopy are for the most part obtained by chance [15, 16]. Thus, the development of reliable and efficient methods to obtain well-dispersed chromosomes with an intact morphology remain a challenge.

The initial aim of this work was to develop an efficient method to prepare high-quality mitotic chromosomes to physically map repetitive DNA sequences in dinoflagellates using traditional FISH. Four naked (without a theca covering the cell) species were selected as a model: *Karlodinium veneficum* and three species of the genus *Karenia*. All four species cause harmful algal blooms and produce toxins that are lethal to fish and other marine organisms [17, 18]. After the failure to obtain a chromosomal preparation suitable for conventional FISH, we developed a simple and effective method that can be used on a cell suspension (FISH-IS), instead of on a microscope slide as usually done. As proof of concept, the FISH-IS approach was successfully used to analyze: 1) dinoflagellate telomere DNA, characterized by repeats of the heptanucleotide 5´-TTTAGG-3´ [12] and detected in this study using (CTTTAAA)_3_ as probe, and 2) the AG-chromosome [15] of *Karenia mikimotoi*, characterized by a particularly high density of AG repeats along its entire length and detected herein using (CT)_10_ as probe.

## Materials and methods

The strains employed in this study, *Karlodinium veneficum* VGO1111, *Karenia mikimotoi* KT77B, *Karenia selliformis* GM94GAB and *Karenia brevis* CCMP2281, are regularly maintained at the Centro Oceanográfico de Vigo (CCVIEO; the Culture Collection of Harmful Microalgae of the Spanish Institute of Oceanography), where they are available upon request.

### Culture conditions

The strains were cultured at 20°C with an irradiance of ~90 μmol photons m^−2s−1^ and a photoperiod of 12:12 h light:dark. Culture stocks were maintained in Iwaki 50-mL flasks filled with 30 mL of L1 medium [19] without added silica. The medium was prepared using Atlantic seawater adjusted to a salinity of 30 psu by the addition of sterile distilled water.

### Culture synchronization

Saturated cell suspensions were harvested by gentle centrifugation (1500 g, 5 min) to remove the L1 medium. The pellet was resuspended in a final volume of 5 mL of L1 medium [19] a 0.2 μg colcemid ((*N*-deacetyl-*N*-methyl-colchicine; Merck-Biochrom)/mL for 3.5 h at 20°C to achieve a high proportion of mitotic cells. The optimal colcemid concentration and incubation time were determined experimentally, by testing concentrations of 0.1 and 0.2 μg/mL and incubations of 2, 3.5 and 5 h and visually checking the number of cells in division.

### Preparation of suspension of chromosomes and nuclei

Suspensions were prepared according to the protocol of the CRG/UPF Flow Cytometry Unit (Barcelona) with minor modifications. Ten mL of a synchronized cell culture was centrifuged for 5 min at 1000 g and room temperature (RT). The supernatant was discarded and the pellet re-suspended in 10 mL of hypotonic solution (HPA: 75mM KCl, 10 mM MgS0_4_, 0.2 mM spermine and 0.5 mM spermidine, pH 8) and incubated for 15 min at RT. To release the chromosomes, the swollen cells were centrifuged for 5 min at 1000 g and RT. The supernatant was discarded and the pellet resuspended in 1.5 mL of ice-cold polyamine isolation buffer (PAB: 15 mM Tris pH 8.0, 1 mM EDTA, 0.5 mM EGTA, 80 mM KCl, 3 mM DTT, 0.25% Triton X-100, 0.2 mM spermine and 0.5 mM spermidine, pH 8) and incubated for 15 min on ice. To liberate the chromosomes, the pellet was vortexed vigorously for 30 s. If necessary, the chromosomes in suspension were separated by vortexing for another 20 s. The suspensions were stored at 4°C until used in slide preparations for FISH or FISH-IS. The yield and morphology of the chromosomes were evaluated by microscopy of Hoechst 33258 (Invitrogen) stained preparations.

### Slide preparation for FISH

Ten μL of the suspension prepared as described above was flattened between a slide and coverslip. After removal of the coverslips by freezing, the slides were air-dried. The samples were incubated with RNase A, fixed in 4% (w/v) paraformaldehyde, dehydrated in a graded ethanol series and finally air-dried, as previously described [20].

### Probes and labeling

The four probes selected for this study were those previously applied to analyze the genus *Karenia* by conventional FISH [15]. The pTa71 probe was used to map the ribosomal genes. This plasmid, which contains a 9-kb *Eco*RI fragment from *Triticum aestivum* that includes the 45S rDNA region, was labeled with digoxigenin-11-dUTP using the Dig-Nick translation mix kit from Sigma-Aldrich. Two probes, (AG)_10_ and (CT)_10_ with biotin and FITC, respectively, incorporated at both ends (Isogen Life Science), were used to visualize the AG-chromosome. The oligonucleotide (CCCTAAA)_3_, synthesized with Dy547 (red) at both ends (Isogen Life Science), was used as the probe for the detection of telomeric repeats.

### FISH

FISH was performed as recently reported [14]. A hybridization solution containing 50% deionized formamide, 10% dextran sulfate, 2×SSC (300 mM sodium chloride, 0.3 mM trisodium citrate), 0.33% SDS, 50 ng of the digoxigenated pTa71 probe and 2 pmol of the biotinated (AG)_10_ probe was denatured in an oven for 10 min at 70°C and 30 μL, then used to flush the sample-bearing slides. The cells were then denatured by placing the slides in an incubator at 75°C for 7 min, with the temperature controlled using a programmable thermal controller (PT-100, M.J. Research). Hybridization was performed by incubating the slides overnight in a humidified chamber at 37°C. The slides were then washed for 10 min in 4× SSC/Tween^20^. Digoxigenin and biotin were detected by incubating the slides in fluoresceinated anti-digoxigenin (Roche Applied Science) and streptavidin-Cy3 (Sigma-Aldrich), respectively, in 5% (w/v) bovine serum albumin for 1 h at 37°C. The slides were then rinsed for 10 min in 4× SSC/Tween^20^ at RT, stained with the DNA stain DAPI (4´, 6-diamidino-2-phenylindole) and mounted in antifade solution (Vectashield, Vector Labs, Curlingame, CA).

### FISH in suspension (FISH-IS)

The centrifugation steps used in the following to pellet the cells were consistently conducted at 1000 g for 10 min. FISH-IS was performed as follows. The supernatant obtained from 100 μL of suspended chromosomes in PAB was discarded and the pellet resuspended in 20 μL of 45% glacial acetic acid for 3.5 min. The pellet was washed by resuspending it after centrifugation in 100 μL of 2×SSC. The cells were labeled by resuspending them in 30 μL of 2×SSC containing 2 pmol of the fluorochrome-labeled oligonucleotide probes: (CT)_10_, (CCCTAAA)_3_ or both. Hybridization was performed by incubating the samples in the dark for 1 h at RT. Then, without further washing or centrifugation, 10 μL of the chromosome suspension was dropped onto a slide and mixed with 3 μL of DAPI (2 μg/mL) for DNA staining and 7 μL of an antifade solution (Vectashield, Vector Labs, Curlingame, CA) for mounting. The chromosomes were then examined using fluorescence microscopy.

### Microscopy and imaging

Microscopic analyses were conducted using an epifluorescence Axiophot Zeiss system. Images were captured with a cooled CCD camera (Zeiss) and merged using Adobe Photoshop. The images were optimized for best contrast and brightness using the same program, but only those functions that treated all pixels in the image equally.

## Results

### Chromosome suspension

**Fig 1:**
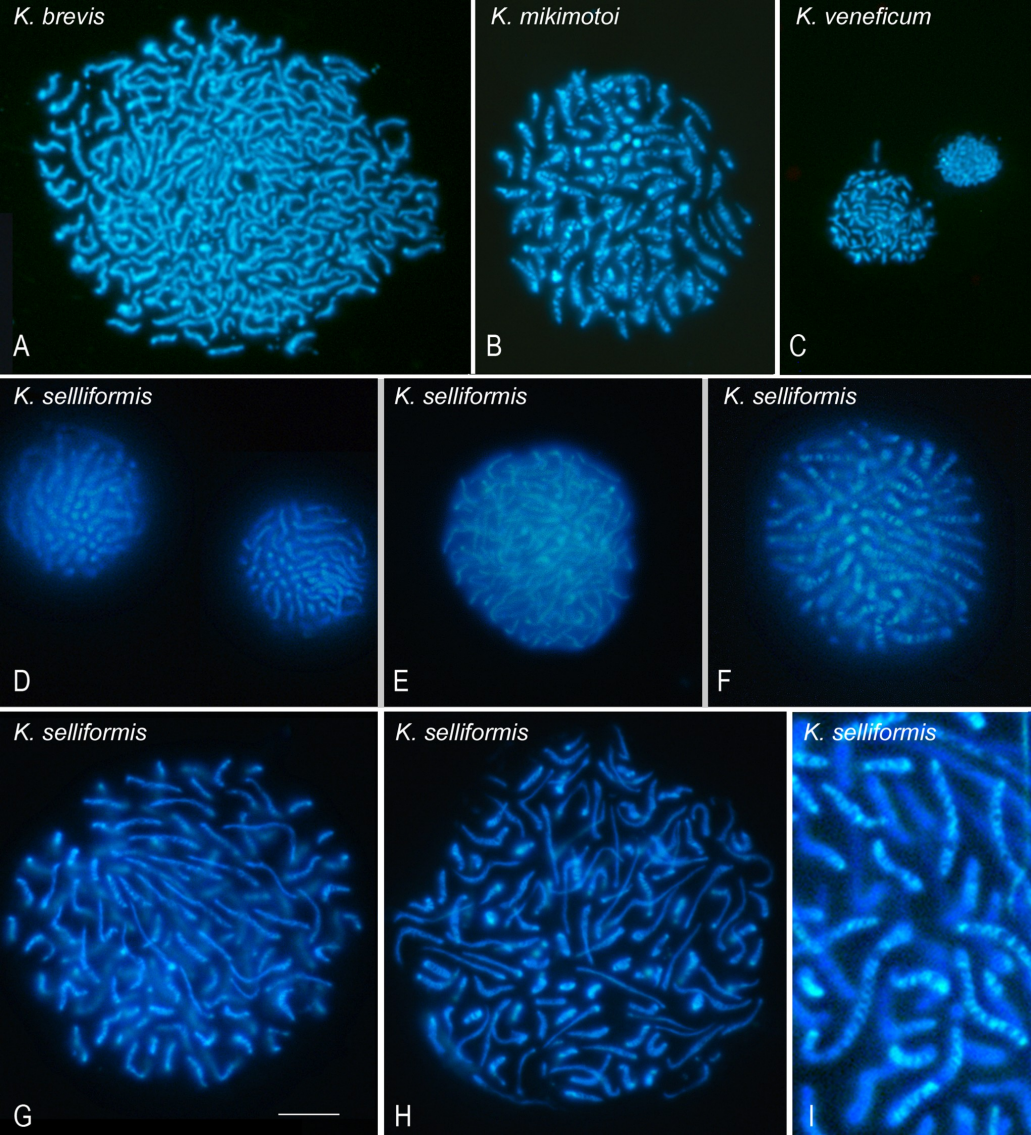
The chromosomal preparation after optimization of the protocol to isolate nuclei in suspension. The nuclei are stained with Hoechst 33258 (blue) (A) *Karenia brevis*, (B) *Karenia mikimotoi*, (C) *Karlodinium veneficum*, *and* (D–I) *Karenia selliformis* nuclei at different stages of interphase (D–F) or during division (G–H). (I) A set of chromosomes at 2× magnification. By individually following some of the chromosomes, their bands can be counted. Scale bar = 10 μm.

In the first series of experiments, the conditions for the preparation of high-quality suspensions of dinoflagellate chromosomes to be used in the FISH experiments were defined (Fig 1). After culturing the microalgal cells under optimal conditions, their mitotic arrest was induced by incubation in 0.2 μg colcemid/mL for 3.5 h, which inhibited cell growth. We then evaluated different methods to achieve the release of the chromosomes from the synchronized dinoflagellate cells, finally choosing a 15-min incubation in hypotonic solution at RT to swell the cells, followed by centrifugation for 5 min and resuspension of the pellet in lysis buffer for 15 min on ice to release the chromosomes. The detergent in the buffer destabilizes the cell membranes while the polyamides stabilize the chromatin, ensuring the preservation of chromosome morphology. Fig 1 shows the results of this process in the four species analyzed. The nuclei of cells from the *Karenia* species and *Karlodinium veneficum* differ greatly in size, with those of *Karenia brevis* (Fig 1A) being much bigger and containing a higher number of chromosomes than nuclei of *Karenia selliformis* (Fig 1D and 1E) and, especially, of *Karenia mikimotoi* (Fig 1B). The nuclei of *Karlodinium veneficum* were the smallest among the four species, as were both its chromosomes and the number thereof (Fig 1C).

Although the nucleosome-less chromosomes of dinoflagellates are defined as permanently condensed, given their consistent appearance throughout the cell cycle as well-defined entities, on closer inspection different degrees of nuclear compaction and chromosome release are observed, depending on the cell-cycle stage. For example, as shown in Fig 1D–1H, the morphology of *K*. *selliformis* nuclei differed over the course of the cell cycle. We accordingly defined interphasic nuclei as those with smaller nuclear areas and chromosomes distributed close to each other (Fig 1D–1F). Mitotic nuclei were those in which the chromosomes were highly separated (Fig 1G and 1H). To obtain a high yield of mitotic nuclei and a high-quality chromosomal suspension, colcemid was added to the culture medium. In the mitotic nuclei, hundreds of chromosomes of different sizes were distinguishable (Fig 1G and 1H). Since their chromatin is not well-preserved by fixation, depending both on the pressure used to spread the chromosomes between the coverslip and slide for microscopy analysis and the position of the chromosomes in the nuclei, some chromosomes were elongated and thinner than others (Fig 1G and 1H). Hoechst 33258 staining was used to monitor the nuclei and the morphology of the subsequently released chromosomes. Unexpectedly, staining resulted in brightly fluorescent telomeres and other interstitial bands. Thus Hoechst staining, which when bound to DNA emits a blue fluorescence, produced a novel, previously undescribed banding pattern in the four dinoflagellate species analyzed (Fig 1B and 1H). A higher magnification of a few chromosomes of *K*. *selliformis* is provided in Fig 1I to better show the Hoechst bands.

### FISH

**Fig 2:**
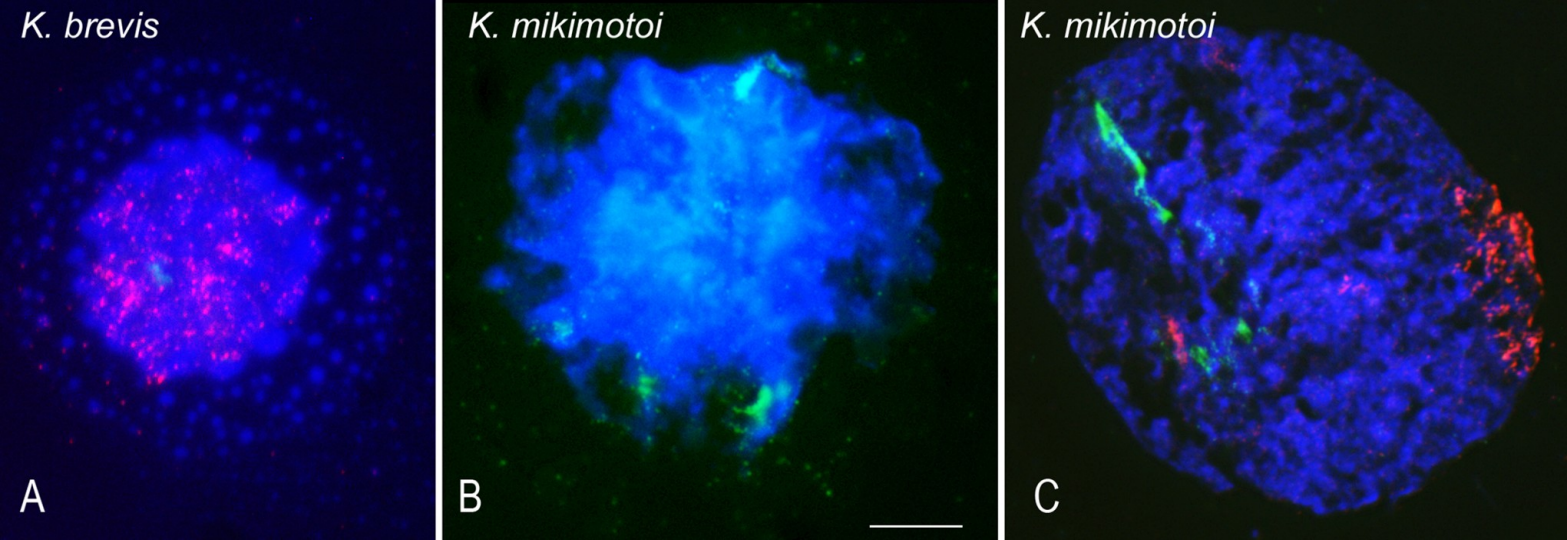
FISH mapping of different repeat sequences in nuclei isolated using the chromosome isolation procedure. (A) *K*. *brevis* and (B, C) *K*. *mikimotoi*. DAPI-stained DNA (blue) and *in situ* hybridization of the 45S rDNA digoxigenin-labeled probe (green) in combination with (A) the Dy547-labeled telomeric probe (red) or (C) the biotin-labeled (AG)_10_ probe (red). Note the largely disturbed nuclear morphology after FISH. Scale bar = 10 μm.

Given that the initial aim of this work was to achieve high-quality chromosomes suitable for the analysis of repetitive DNA sequences using traditional FISH, we first tested different protocols to spread and adhere chromosomes in suspension onto the slide surface. The best results were obtained using the squashing rather than the equally common air-dry dropping method of slide preparation, as the former reduced the amount of loss of nuclei and chromosomes after FISH. After a pre-treatment to preserve chromosomal morphology and increase the efficiency of the hybridization, the FISH protocol successfully used for ~25 years in our lab in different species, including dinoflagellates, was applied. However, it altered the chromatin structure of the suspended chromosomes and the hybridization results were poor (Fig 2). Most of the nuclei were not intact and the chromatin was diffuse. In many nuclei, the chromosomes had lost their typical appearance; instead, the expanded DNA appeared as concentric circles of blue DAPI bubbles differing in size around a still-intact central area. For example, compare the chromatin structure of the nuclei of *K*. *brevis* (Fig 1A) before FISH and a nucleus of the same specie after FISH using the 45S rDNA (green) and telomeric (red) probes (Fig 2A). Also common were nuclei with disrupted chromatin, which complicated correct assessments of the number of hybridization signals. Fig 2B shows a nucleus of *K*. *mikimotoi* after FISH using the 45S rDNA probe. Instead of the typical discrete clusters of hybridization signals, both a nebulae of dots, indicative of the dispersed state of the chromatin, and extruded fibers around the nuclear boundary were seen. Thus, while the resolution achieved in the disrupted nuclei was sufficient to co-localize different probes using two-color FISH (Fig 2A and 2C), valuable information regarding the precise number of loci and the locations of the repeat sequences analyzed was lost. Clearly, high-quality sample preparation is a prerequisite of efficient and reliable cytogenetic investigation. Conventional FISH is an aggressive technique that destroys the morphology of nuclei and chromosomes preserved with the protocol used in our study to isolate unfixed chromosomes in suspension. This can be observed by comparing the morphology of the nuclei shown in Fig 1, prepared after applying the protocol based on polyamine-mediated chromosome isolation to obtain chromosomes in suspension, and those shown in Fig 2, after FISH.

### FISH-IS

**Fig 3:**
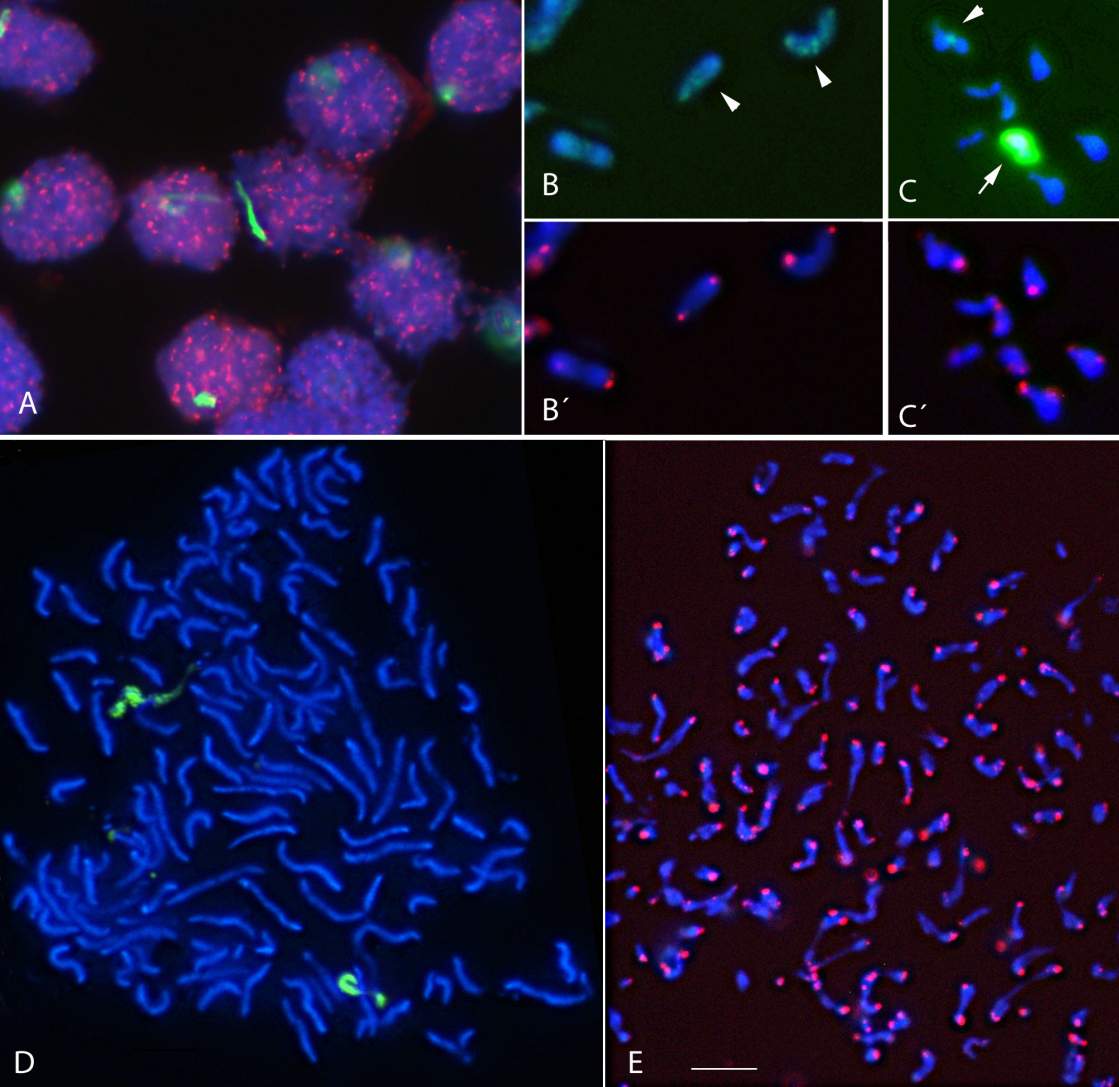
Images of FISH in suspension (FISH-IS) using the isolated nuclei and chromosomes of *Karenia mikimotoi*. DAPI-stained DNA (blue) and *in situ* hybridization of the Dy547-labeled oligonucleotide (CCCTAAA)_3_ used to localized telomeric repeats (red) (A, B´, C´ and E) and the FITC-labeled oligonucleotide (CT)_10_ used to paint the AG-chromosome (green) (A, B, C and D) in interphasic nuclei (A), isolated chromosomes (B, C) and mitotic nuclei with different degrees of chromosomal condensation (D, E). Arrowheads in B and C point to interstitial clusters of CT repeats. The arrow in C points to the strongly labeled AG-chromosome. Scale bar = 10 μm.

As an alternative to traditional FISH, in situ hybridization can be performed in suspension. Given the above-described failure of conventional FISH, we sought to develop a reliable FISH-IS method for the analysis of repetitive DNA on morphologically preserved chromosomes in suspension. Using two fluorescence oligonucleotide probes targeting the telomeres and the AG microsatellite, respectively, we were able to convincingly demonstrate that the complete double-target FISH procedure can be performed in a suspension of chromosomes obtained from the dinoflagellate *Karenia mikimotoi* (Fig 3).

A prerequisite for our novel FISH-IS method is denaturation of the DNA double helix by treating the chromosome suspensions with 45% acetic acid, which contributes to preserving chromosomal morphology. After 3.5 min, the cells are pelleted by centrifugation and then washed in 2×SSC. The distinctive accumulation of the microsatellite AG in one specific chromosome of *K*. *mikimotoi* identifies and defines the AG-chromosome, which can be efficiently painted in interphasic nuclei (Fig 3A) as well as on released chromosomes (Fig 3B–3D) using the (CT)_10_ oligonucleotide labeled with FITC (green) as probe. Clusters of microsatellite CT scattered in other chromosomes were also observed, but only in chromosomes sufficiently isolated from the stronger FISH signals obtained from the AG-chromosome (arrows in Fig 3C), which otherwise masked weaker signals dispersed over the other chromosomes (arrowheads in Fig 3B and 3C). Dinoflagellates share with plants a simple repeat of the heptanucleotide 5´-TTTAGGG-3´ as a telomeric sequence. The Dy547-labeled (red) oligonucleotide (CCCTAAA)_3_ is highly effective in detecting telomeres both in interphasic nuclei (Fig 3A) and at the end of chromosomes in suspension (Fig 3B, 3C and 3E). Moreover, hybridization was achieved within a 1-h incubation at RT; thus FISH-IS can be completed in less than 90 min. In addition, the chromosomal suspension can be stored at 4°C, extending the time until the FISH-IS analysis to one month, at least.

## Discussion

### Studying chromosomes of dinoflagellates

Unlike eukaryotes, in which the maximum degree of compaction is reached during metaphase, in dinoflagellates, the chromosomes lack nucleosomes and remain well-defined entities during both interphase and mitosis (last review in [5]). A prerequisite to understanding chromosomal organization and function in dinoflagellates was to develop a relatively simple method of chromosome preparation [10]. Most modern molecular cytogenetics techniques, such as FISH, require well-spread and morphologically intact chromosomes. However, because dinoflagellate chromosomes remain in close proximity even during mitosis and are permanently surrounded by the nuclear envelope, previously established methods of preparation, including those for light microscopy, are ineffective. A modification of the squashing method used in plants has been the common procedure for FISH chromosome preparation in dinoflagellates [12, 14, 15] but it mostly relies on the chance extrusion of individual chromosomes from squashed cells.

The first requirement of our FISH-IS method was to optimize the isolation of chromosomes from synchronized cells. As noted above, dinoflagellate chromosomes remain condensed during the entire cell cycle (G1, S, G2 and M); however, transient unwinding during genome replication in S phase was presumed, as experimental evidence has clearly shown that transcription in dinoflagellates occurs on extrachromosomal DNA filaments protruding from the cores of their chromosomes [21]. This conclusion received further support from electron microscopy studies showing numerous structures protruding toward the nucleoplasm of dinoflagellate nuclei in G1 [11]. While yet to be confirmed experimentally, it seems likely that nuclei with the closest and most interconnected chromosomes are those that form these temporary decondensed loops or foci of unwinding in interphase, while nuclei undergoing division are those in which the chromosomes are separated.

In this work, a high index of mitotic chromosomes was obtained using colcemid to the culture medium. By inhibiting the polymerization of tubulin, the major component of microtubules, the development of functional mitotic spindles was inhibited as well. In dinoflagellate mitosis, chromosomes that are attached to the inner aspect of the nuclear membrane are in contact with the mitotic spindle assembled outside the nucleus, through cytoplasmic invaginations that cross the nucleus to form channels containing microtubular bundles [22]. Thus, the metaphase-like nucleus of dinoflagellates is identifiable by the presence of cytoplasmic channels [11, 15]. Our protocol to prepare chromosomes included the use of a lysis buffer to break up membrane structures, including the cytoplasmic channels. Accordingly, we defined a metaphase-like stage morphologically, based on the presence of an enlarged nucleus with a relatively high degree of chromosomal separation (Fig 1A–1C, 1G and 1H). In this study, we did not rigorously evaluate the effect of different colchicine treatments during the dinoflagellate cell cycle [23], as it sufficed to visually determine the optimal concentration and exposure time needed to obtain chromosomal preparations with a quality appropriate for FISH analysis. Although the chromosomes of nuclei identified as interphasic remained close together, the addition of a hypotonic solution caused the dividing cells to swell, whereas the addition of a buffer containing detergent and polyamines destabilized the membranes, thus releasing the chromosomes. With this method, we were able to successfully obtain high-quality chromosomes in suspension. Furthermore, the long storage time of the cell suspensions did not impair the quality of the chromosomal preparations subsequently used for FISH-IS.

### Dinoflagellate chromosome differentiation

The chromosomes of dinoflagellates do not show longitudinal differentiation. Indeed, a distinctive feature is their lack of a chromosomal constriction formed by centromeres/kinetochores. In typical eukaryote chromosomes, these sites define the tightly packed chromatin region that joins sister chromatids and attaches them to the spindle during mitosis [24]. In addition, dinoflagellate chromosomes do not exhibit the characteristic and reproducible transverse banding patterns, such as the Q- G- or C-bands, of stained eukaryotic chromosomes. In the latter, those bands are used in the longitudinal differentiation and in the identification of specific chromosomes [7, 25]. Nevertheless, under some conditions, phase-contrast microscopy reveals a longitudinal, periodic striation at the surface of chromosomes that are in different degrees of spreading. This pattern has been suggested to correspond to the presence of double helicoidal bundles of filaments that are well resolved on electron microscopy [24, 26]. A correspondence between the above-described, brightly fluorescent, more or less transverse bands seen over the length of Hoechst-stained chromosomes and the helical arrangement of coiled chromosomes observed on electron micrographs seems likely. However, like DAPI, the Hoechst dye binds to the minor groove of double-stranded DNA, with a preference for sequences rich in adenine and thymine. If not indicative of a hallmark of the dinoflagellate genome, then, at the very least, the Hoechst staining pattern shows that the genomes of the four species analyzed in this study are not uniformly organized, but instead contain clusters of AT-rich DNA sequences interspersed over the length of each chromosome. However, very little is known thus far about how dinoflagellate DNA is organized and packed into functional chromosomes [27]. Investigations of this important question are needed.

### FISH

When analyzed under the microscope, our suspension method yielded chromosomes that, after being flattened on the slide surface, had an intact morphology. However, after the FISH procedure both the nuclear and the chromosomal morphology was destroyed, as there were many DNA protuberances emerging from the nuclear periphery. In addition, in some nuclei a DNA halo around the nuclear boundary, which complicated localization of the hybridization signals such that the hybridization results were poor.

This outcome was attributed to the denaturation step, which is the most damaging with regard to nuclear morphology. Nonetheless, denaturation/renaturation is essential to the detection of the target sequences in FISH and it must be preceded by a fixation step to prevent chromosomal disintegration. As was the case in our study, the most common method to denature DNA for FISH is heating in the presence of formamide. Although before FISH we pretreated the slides with 4% paraformaldehyde, a fixative that preserves chromosomes of dinoflagellates previously fixed and conserved with ethanol in conjunction with acetic-acid relatively well [12], fixation with 4% paraformaldehyde did not prevent chromatin damage in dinoflagellate nuclei isolated using a modification of the method used for chromosome sorting in human lymphocytes [28]. Clearly, a suspension of non-fixed dinoflagellate cells adhered onto the slide surface is not resistant to FISH processing even if a fixation treatment with paraformaldehyde before dehydration in a graded ethanol series and air-drying is performed before FISH. The lack of a nucleosomal-type organization and the much lower DNA:protein ratio (10:1) of dinoflagellate vs. typical eukaryote (1:1) chromosomes could account for the above-described loss of DNA [29]. The FISH-IS method developed and successfully used in this study does not involve heat, formamide, or alkaline treatment to denature the target DNAs. Nonetheless, it allowed the detection of bright hybridization signals at the regions of interest of morphologically intact nuclei and chromosomes isolated in solution.

### FISH-IS

Attempts to apply standard FISH procedures to chromosomes in suspension (FISH-IS), both in animals and in plants, have largely failed, in part because under these conditions chromosomes tend to disintegrate or clump when subjected to the heat-based processes required for both chromosome and probe denaturation. In addition, FISH-IS requires solution changes as well as washing and pelleting the chromosomes, which together cause sample losses and a hybridization pattern with poor reproducibility [30, 31]. The FISH-IS protocol developed by Giorgi et al., 2013 [32] has a reduced number of washing and centrifugation steps and uses alkaline DNA denaturation together with oligonucleotide probes to label microsatellite DNA. The method has been tested in chromosomal suspensions prepared from wheat and related species [33]. Our FISH-IS method takes advantage of a protocol to obtain high-quality chromosomal suspensions, an adaptation of a protocol used to sort human chromosomes and the feasibility of using oligonucleotides to detect repeat sequences, and hence specific chromosomes, also in dinoflagellates [15]. Moreover, alkaline denaturation is unnecessary as acetic acid pretreatment suffices to make the chromosomes susceptible to hybridization with fluorescence-labeled oligonucleotides. In the analysis of specific repetitive DNA sequences during karyotyping, oligonucleotides have many advantages over DNA probes, including their small size and thus their better penetration, which increases the sensitivity of signal detection. In addition, because they are single-stranded, they exclude the possibility of probe renaturation [34]. We tested two different labeled oligonucleotides as probes: Dy547-labeled (CTTTAAA)_3_ and FITC-labeled (CT)_10_. After a 1 h incubation of the probes (suspended in 2×SSC) with the cell suspensions at RT, the telomeres and the AG-chromosome of *K*. *mikimotoi* were easily and quickly detected without the need for further washing and centrifugation steps. After an aliquot of the cell suspension is stained with DAPI and mounted in antifade solution between a slide and a coverslip, the sample can be analyzed using epifluorescence microscopy. Our results demonstrate that acetic acid treatment enables DNA denaturation while preserving chromosomal morphology during the *in situ* hybridization [35]. Thus, compared to conventional FISH performed on squashed samples on microscope slides, our novel FISH-IS method allows the unambiguous localization of specific repeat DNA sequences in dinoflagellate cells along with a higher degree of chromosomal separation, and hence of signal resolution.

The new approach to dinoflagellate chromosome analysis offered by FISH-IS will contribute to the analysis of specific chromosomes that can be discerned by specific oligonucleotide probes, such as the AG-chromosome of *K*. *mikimotoi* [15] and the ribosomal chromosomes of some *Alexandrium* species [14]. As more information from the repetitive sequences of chromosomes accumulates, oligonucleotides, which in addition to their superior hybridization kinetics have manufacturing costs lower than those of traditional genomic probes, will become valuable tools for studying chromosomal organization and evolution in dinoflagellates. Our results suggest that the FISH-IS approach can be used to discriminate chromosomes before sorting. However, the ability to sort dinoflagellate chromosomes—and thus differentiate among the hundreds of chromosomes of similar size that cannot be distinguished morphologically—will facilitate investigations into the very large and highly redundant genomes of dinoflagellates [2]. Indeed, flow-sorted chromosomes can be used in applications ranging from physical mapping to genomic studies, for example, to simplify analyses of complex plant polyploid genomes [36].

## Conclusions

By coupling chromosome isolation and FISH, we developed the first technique that allows the labeling of acetic-acid-pretreated dinoflagellate chromosomes in suspension using fluorescence oligonucleotides as probes. Our approach should be applicable to other species of interest for which liquid suspensions of high-quality chromosomes can be prepared and microsatellites or other simple DNA repeat sequences are available for oligonucleotide-based detection.
